# The Role of Vitamins in Sepsis: A Narrative Review

**DOI:** 10.3390/nu17213330

**Published:** 2025-10-23

**Authors:** Paweł Radkowski, Anna Gogojewicz, Joanna Charasna, Łucja Pilaczyńska-Szcześniak, Łukasz Grabarczyk

**Affiliations:** 1 Department of Anaesthesiology and Intensive Care, Faculty of Medicine, Collegium Medicum, University of Warmia and Mazury, 10-719 Olsztyn, Poland; pawel.radkowski@uwm.edu.pl; 2 Clinical Department of Anesthesiology and Intensive Care, Regional Specialist Hospital, 10-561 Olsztyn, Poland; 3 Klinika-Hospital Zum Heiligen Geist, 34560 Fritzlar, Germany; 4 Department of Food and Nutrition, Poznan University of Physical Education, 61-871 Poznań, Poland; 5 Emergency Medical Services Training Center in Kalisz, 62-800 Kalisz, Poland; joanna.charasna@gmail.com; 6 Faculty of Finance and Management, WSB University Merito in Wrocław, 53-609 Wrocław, Poland; 7 Department of Physiotherapy, Faculty of Medicine and Health Sciences, University of Kalisz, 62-800 Kalisz, Poland; pilaczynskal@gmail.com; 8 Alarm Clock Clinic, Coma Recovery and Neurorehabilitation Center, Kondratowicza 8, 03-242 Warszawa, Poland; grabarczyk@gmail.com

**Keywords:** sepsis, vitamin D, thiamine, folic acid, vitamin C, vitamin B12, adjunctive treatment, oxidative stress, immunomodulation, targeted therapy

## Abstract

Sepsis remains one of the major challenges in modern intensive care, characterized by high mortality and complex metabolic and immunological disturbances. Given the limited effectiveness of current therapeutic strategies, increasing attention has been directed toward supportive interventions aimed at restoring metabolic homeostasis. Particular interest has been focused on selected vitamins that exhibit pleiotropic biological effects. Thus, we summarized the current evidence on the role of selected vitamins (C, D, B1, B9, B12) in the treatment and supportive management of sepsis, highlighting their mechanisms of action, potential clinical benefits, and limitations derived from available studies. A comprehensive analysis of the literature was performed, including clinical trials and meta-analyses evaluating the efficacy of vitamin supplementation in sepsis, with particular emphasis on combined interventions and randomized controlled trials in severe sepsis and septic shock. Vitamin D might demonstrate the greatest therapeutic potential, particularly in patients with severe sepsis and respiratory failure, with benefits associated with achieving appropriate therapeutic concentrations. Thiamine (vitamin B1) appears to provide potential advantages primarily in deficient patients, improving mitochondrial function and reducing the risk of renal failure. Evidence regarding folic acid (vitamin B9) and cobalamin (vitamin B12) remains inconclusive, as both deficiency and elevated serum levels have been linked to adverse outcomes. Vitamin C, despite its well-documented antioxidant and microcirculatory effects, has not yet shown consistent evidence of mortality reduction. In conclusion, current evidence suggests that vitamin supplementation might represent an adjunct to standard sepsis therapy, particularly within a personalized approach that considers nutritional status and metabolic phenotype. The development of standardized dosing protocols and well-designed clinical trials is essential to determine the efficacy and safety of phenotype-driven individualized approaches in sepsis management.

## 1. Introduction

Sepsis, defined as life-threatening organ dysfunction caused by a dysregulated host response to infection, represents one of the most serious clinical challenges of the 21st century. According to the 2020 report by the World Health Organization, more than 50 million cases of sepsis occur globally each year, with approximately 11 million resulting in death—accounting for nearly 20% of all deaths worldwide [1]. Despite advances in intensive care medicine, antibiotic therapy, modern fluid resuscitation strategies, and sophisticated monitoring techniques, the mortality rate associated with sepsis—particularly septic shock—remains alarmingly high. According to data from the Surviving Sepsis Campaign, mortality in intensive care units may reach as high as 40–50% [2]. Sepsis, as defined by Sepsis-3 criteria developed by the SCCM (Society of Critical Care Medicine) and ESICM (European Society of Intensive Care Medicine), is a life-threatening organ dysfunction caused by a dysregulated host response to infection. It is most commonly identified by an increase in the SOFA (Sequential Organ Failure Assessment) score of at least 2 points [3]. Attempts to use SIRS (Systemic Inflammatory Response Syndrome) criteria for early diagnosis have proven less accurate, highlighting the need for precise diagnostic tools and effective monitoring [4]. Recent data from middle- and high-income countries show a growing number of hospitalizations due to sepsis, linked to factors such as an aging population, an increasing number of invasive procedures, and the widespread use of immunosuppressive therapies [5]. Although most patients with sepsis survive hospitalization, studies show that 28-day mortality ranges from 24% to 30%, while septic shock results in death in as many as 40–50% of cases [2]. Moreover, long-term observations indicate that the average mortality rate among sepsis patients reaches 32–33% within three years, exceeding rates observed in other severe inflammatory conditions [6]. Sepsis is characterized by a complex clinical course and a diverse spectrum of symptoms, including fever or hypothermia, tachycardia, tachypnea, low blood pressure, altered mental status, oliguria, as well as disturbances in microcirculation and coagulation [3]. Key assessment tools include the SOFA and qSOFA scores, although the latter serves only as a diagnostic aid [4]. From a pathophysiological perspective, sepsis results from an extreme inflammatory response accompanied by immune dysregulation, endothelial damage, activation of the coagulation system, and progressive organ ischemia [3]. Within the framework of the Sepsis-3 definition, distinct patient subphenotypes have been identified—dominated by inflammation, immunosuppression, or microcirculatory dysfunction—which suggests that the future of diagnostics may lie in accurately identifying these subgroups [6]. Given the limited effectiveness of conventional therapies, there is growing interest in adjunctive treatment strategies for sepsis that could positively modulate the immune response, reduce oxidative stress, and support metabolic processes in critically ill patients. One area of research that has gained particular attention in recent years is the use of micronutrients and vitamins—especially vitamin C, thiamine (B_1_), and vitamin D—as potential adjuvant therapies. As early as 2017, a study by Marik et al. [7] sparked widespread discussion in the medical community, suggesting dramatic clinical benefits from a combined therapy using vitamin C, thiamine, and hydrocortisone in patients with sepsis. Although initial reports were promising, subsequent randomized clinical trials—including VITAMINS, ACTS, CITRIS-ALI, and LOVIT—produced results that were far more modest or even contradictory [8,9,10]. Recent systematic reviews and meta-analyses indicate that while certain clinical parameters may improve with the supplementation of selected vitamins, the evidence for mortality reduction remains insufficient, and the results of individual studies are marked by high heterogeneity [11]. Moreover, concerns have been raised about potential adverse effects of intensive vitamin therapy, particularly in cases of improper dosing or inadequate patient selection. In light of these findings, the use of vitamins in the treatment of sepsis remains controversial and calls for further well-designed clinical trials. On the one hand, we are dealing with inexpensive and widely available substances with potential protective effects; on the other, there is a lack of clear clinical evidence to justify their routine inclusion in therapeutic standards. This article aims to provide a critical analysis of the current state of knowledge in this field and to evaluate the potential role of vitamins as adjunctive therapy in the treatment of sepsis.

## 2. Materials and Methods

This narrative review was conducted through a systematic and thematic synthesis of peer-reviewed literature published between January 2016 and July 2025. A comprehensive literature search was conducted in PubMed, Scopus, and Web of Science. Search strategies included combinations of controlled vocabulary and free-text terms related to sepsis, vitamin-based adjunctive therapies (e.g., vitamin D, thiamine, folic acid, vitamin C, vitamin B12), and relevant biological mechanisms such as oxidative stress, immuno-modulation, and targeted therapy. Boolean operators (AND, OR) were applied to refine the search. Reference lists of key articles and meta-analyses were manually screened to identify additional relevant studies. Studies were included based on the following criteria: published in English, focused on adult patients diagnosed with sepsis or septic shock, investigated the use of vitamins C, D, B1 (thiamine), B9 (folic acid), or B12 as adjunctive therapies, and reported either clinical outcomes, mechanistic insights, or biomarker data. Excluded studies were: animal or in vitro studies without translational relevance, case reports or expert opinions without empirical data, and studies with incomplete methodology or lacking peer review.

The review was structured thematically, and data were analyzed in a descriptive approach to examine the benefits, mechanisms, and intervention. Recommendations were synthesized based on the strength of available evidence, clinical relevance, and feasibility of long-term adherence. Where applicable, findings were contextualized within current guidelines (e.g., Surviving Sepsis Campaign) and emerging frameworks for precision medicine in critical care.

## 3. The Therapeutic Potential of Vitamins in Sepsis Management

### 3.1. Vitamin C

Vitamin C (ascorbic acid), owing to its antioxidant and immunomodulatory properties, as well as its role in supporting endogenous catecholamine synthesis, has been widely investigated as a potential adjunctive therapy in sepsis. Initial enthusiasm—fueled by protocols such as HAT (high-dose vitamin C, hydrocortisone, and thiamine) and encouraging findings from early observational studies—has gradually shifted toward more cautious interpretation, as results from large, controlled trials have yielded inconsistent outcomes.

Among the most recent and significant studies is the multicenter, prospective, double-blind phase 3b randomized controlled trial “C-EASIE”, conducted across eight hospitals in Belgium. Patients with sepsis or septic shock received 1.5 g of intravenous vitamin C every 6 h for 4 days, with treatment initiated within 6 h of admission. The primary endpoint was the change in SOFA score between days 2 and 5, which, although 8.7% lower in the vitamin C group (1.98 vs. 2.19), didn’t reach statistical significance (*p* = 0.30). However, a planned subgroup analysis of patients with an initial SOFA score ≥ 6 revealed a significant reduction in organ dysfunction (rate ratio 0.76; *p* = 0.042), and a per-protocol analysis showed a decreased need for renal replacement therapy (RRT) (rate ratio 0.28; *p* = 0.05) [12]. In the biological substudy of the LOVIT trial conducted by Rynne et al. [13], no clear anti-inflammatory effect was observed among sepsis patients whose inflammatory markers were analyzed (n = 457). However, a distinct heterogeneity of effect was identified depending on the inflammatory phenotype, underscoring the need for more precise patient selection for therapy. The primary LOVIT trial (Lessening Organ Dysfunction with Vitamin C) enrolled 872 hospitalized patients with sepsis across 35 centers in Canada, France, and New Zealand. Of these, plasma samples were available for 457 participants (53%) at baseline and again seven days after randomization. In this substudy, Rynne et al. [13] hypothesized that different sepsis subtypes may respond differently to high-dose vitamin C therapy, employing cluster analysis to identify biological phenotypes. In the initial phase, an agglomerative cluster analysis was conducted based on log-transformed (log_10_) values of 26 pro- and anti-inflammatory markers collected on day 0, which enabled the identification of three distinct patient groups. Subsequently, logistic regression with robust standard error estimation was used to compare treatment effects (vitamin C vs. placebo) with respect to the primary outcome—death or persistent organ dysfunction by day 28. The analyses were conducted in a blinded manner, in accordance with the study protocol, eliminating any influence of treatment allocation knowledge on result interpretation.

The findings revealed that although no beneficial effect of vitamin C was observed in the overall cohort (OR ≈ 1), the differences between subtypes were statistically significant (*p* = 0.002, heterogeneity test). Subtype 1 (moderate levels of markers) had an OR = 1.04 (95% CI 0.63–1.73), subtype 2 (most pronounced inflammatory state) had an OR = 1.33 (0.53–3.36), and subtype 3 (lowest inflammatory state) had an OR = 1.95 (0.85–4.49), indicating a trend toward potential harm in the latter group. An analysis of timing and interaction with hydrocortisone showed that changes in inflammatory markers depended on the timing of administration and concurrent corticosteroid treatment, with no clear anti-inflammatory effect attributable to vitamin C alone [13].

In a retrospective analysis of data from the MIMIC-IV database, He and Liu [14] conducted a comprehensive study aimed at evaluating the association between vitamin C administration and clinical outcomes in patients with sepsis-associated acute kidney injury (SA-AKI). The study included 16,140 adult patients admitted to intensive care units at Beth Israel Deaconess Medical Center between 2008 and 2019, all of whom met the diagnostic criteria for sepsis and acute kidney dysfunction.

Among this population, 589 patients received intravenous vitamin C as part of standard treatment. To minimize the influence of confounding variables and enhance the reliability of therapeutic effect estimates, advanced statistical methods were applied, including multivariable Cox regression, overlap weighting using propensity scores, and propensity score matching (PSM). The variables included in the analysis encompassed age, sex, disease severity scores (SOFA, SAPS II), the need for renal replacement therapy, use of mechanical ventilation, laboratory parameters, and the presence of comorbidities. The results of the analysis demonstrated that vitamin C supplementation was significantly associated with reduced in-hospital mortality—the adjusted hazard ratio (aHR) was 0.67 (95% CI: 0.57–0.79; *p* < 0.001), corresponding to a 17% reduction in the risk of death compared to the control group not receiving vitamin C. This beneficial effect persisted regardless of the statistical model applied and remained consistent across sensitivity analyses. Kaplan–Meier survival curves further revealed a significant separation in survival trajectories be-tween the groups, confirming notable differences in time to death. Moreover, subgroup analysis did not demonstrate significant interactions between treatment effect and demographic or clinical characteristics such as age, sex, SOFA score, need for mechanical ventilation, or use of renal replacement therapy, suggesting a potentially broad applicability of this intervention. Although the retrospective nature of the study precludes definitive causal inference, and the lack of data on timing and dosage of vitamin C administration limits full interpretation of the findings, the results provide an important signal indicating potential benefits of this intervention in a specific population of critically ill patients. The authors emphasize the need for randomized controlled trials with a well-defined cohort of patients with SA-AKI to confirm the observed as-sociations and to develop effective therapeutic strategies [6].

Findings from previous randomized controlled trials (RCTs), such as CITRIS-ALI [8], VICTAS [15], and LOVIT [10], didn’t confirm the benefits of high-dose intravenous vitamin C administration in patients with sepsis. Some analyses even suggested a potential increase in the risk of organ dysfunction or death, particularly in cases where treatment was initiated with a delay. For example, in the LOVIT trial analysis, an increased risk of death and persistent multiorgan dysfunction was observed in the group treated with ascorbate, which was partially attributed to the short duration of therapy and the abrupt discontinuation of the drug.

In contrast to earlier findings, more recent studies from 2024–2025 [12,14] offer a refreshed perspective, placing particular emphasis on the timing of treatment initiation, patient characteristics, and dosing strategies. The randomized controlled trial “C--EASIE” demonstrated [12] that early administration of vitamin C (within 6 h of admission) resulted in reduced organ dysfunction among high-risk patients (SOFA ≥ 6), although the overall effect was not statistically significant. A retrospective analysis of the MIMIC--IV database further supported the notion that vitamin C supplementation may reduce in-hospital mortality, particularly in patients with acute kidney injury [14].

**Figure 1:**
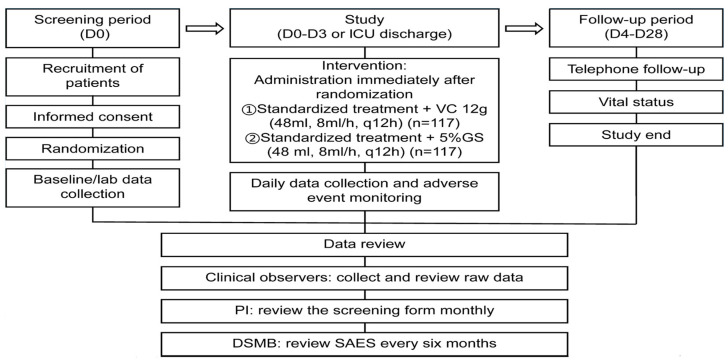
Clinical trial procedure of the CEMVIS study. Participants received intravenous vitamin C (VC) diluted in 5% glucose solution (GS) every 12 h (q12h). The study was overseen by the principal investigator (PI) and monitored by the Data and Safety Monitoring Board (DSMB) [15].

The CEMVIS study (Clinical Efficacy of Megadose Vitamin C in Sepsis) is a multi-center, randomized, single-blind, placebo-controlled clinical trial designed to evaluate the efficacy of megadose vitamin C therapy in patients with sepsis. The study enrolled 234 adult patients aged 18 to 80 years, hospitalized in intensive care units across four clinical centers in China. Inclusion criteria required fulfillment of the Sepsis--3 definition and an elevated procalcitonin (PCT) level of at least 2 ng/mL. Patients were randomly assigned to one of two groups: The intervention group received 12 g of intravenous vitamin C every 12 h for up to 4 days or until ICU discharge, while the control group received an equivalent volume of 5% glucose solution as a placebo. The primary endpoint of the study is 28-day mortality. Secondary endpoints include assessment of organ function (including pulmonary oxygenation parameters), levels of inflammatory markers, duration of hospitalization, and the frequency and severity of adverse events. High-dose vitamin C is administered to reach therapeutic plasma levels of ascorbate, which may help reduce oxidative stress, support mitochondrial function, and modulate the inflammatory response. The study builds upon earlier pilot observations that suggested mega doses of vitamin C could enhance ventilatory parameters and reduce levels of pro-inflammatory cytokines, such as interleukin-6. The authors of the protocol hypothesize that rigorous selection of the study population, a precisely defined dosing regimen, and strict clinical monitoring will yield reliable data that could potentially reshape the approach to sepsis treatment using vitamin C. As of now, the trial remains ongoing, with completion and publication of results expected no earlier than 2025 [16]. The CEMVIS project has been approved by the ethics committee of Zhujiang Hospital of Southern Medical University (approval no. 2020--KY--069--05), and its protocol has been published in The Journal Intensive Care Research [Figure 1].

Based on the available data, routine use of vitamin C in all patients with sepsis cannot currently be recommended. However, evidence from recent analyses suggests that with appropriate patient selection, early initiation, and monitored administration, clinical benefits may be achievable—particularly in patients with SA-AKI. The context of personalized therapy is gaining particular importance; future studies may help identify which patient subgroups derive the greatest benefit from such an intervention.

### 3.2. Thiamine

Thiamine (vitamin B1) plays a crucial role in cellular energy metabolism, serving as an essential cofactor for the enzymatic complexes pyruvate dehydrogenase and α-ketoglutarate dehydrogenase, which are involved in the Krebs cycle. During sepsis, mitochondrial dysfunction occurs, accompanied by a shift in cellular metabolism towards anaerobic glycolysis. This leads to excessive lactate production and exacerbation of metabolic acidosis. Clinical studies indicate that thiamine deficiency affects a substantial number of patients with severe sepsis or septic shock, including both adults and children. Total thiamine concentrations in these patients often fall below reference values (e.g., <8 nmol/L), and deficiency correlates with more severe disease progression, increased lactate production, and a higher likelihood of requiring renal replacement therapy.

A review of recent studies indicates that thiamine monotherapy may be particularly beneficial in selected groups of patients with laboratory-confirmed deficiency of this vitamin. In a post hoc analysis of pooled data from two randomized clinical trials involving a total of 158 patients with septic shock, thiamine administration significantly increased the likelihood of survival without the need for renal replacement therapy (adjusted OR 2.05; 95% CI 1.08–3.90) [9]. Notably, among patients with documented thiamine deficiency, this effect was even more pronounced—aOR 8.17 (95% CI 1.79–37.22)—with a statistically significant interaction (*p* = 0.016), underscoring the importance of patient selection based on vitamin B_1_ levels [17].

In a meta-analysis of five randomized trials evaluating thiamine monotherapy (total n ≈ 300), no significant differences in mortality were observed (RR 0.87; 95% CI 0.65–1.16), although favorable trends were noted in metabolic parameters, such as reduced lactate levels and improved acid-base balance [18]. The study by Petsakul [19] and a systematic review and meta-analysis [20] provided additional data, indicating beneficial metabolic effects and improved renal function, although they did not demonstrate an impact on primary endpoints such as in-hospital mortality. In a systematic review and meta-analysis conducted by Sangla et al. [18] and published in Frontiers in Medicine, five randomized clinical trials (n = 293) were evaluated in which thiamine was administered without co-administration of vitamin C. The analysis showed no significant effect on 28-day mortality (RR 0.87; 95% CI 0.65–1.16; *p* = 0.34), although favorable metabolic trends were observed, such as reductions in lactate levels. The authors noted, however, that the studies were too small to rule out a clinically meaningful benefit and emphasized the need for well-designed, large-scale prospective trials [18].

In the context of combination therapy (vitamin C + thiamine + hydrocortisone), known as the “HAT protocol”, randomized trials such as VITAMINS, ACTS, VICTAS, and BIHAR have demonstrated no advantage of this approach over the use of gluco-corticoids alone [15]. Several of these studies reported no significant differences in survival, vasopressor-free days, or duration of mechanical ventilation. Moreover, some observational analyses of large patient cohorts suggest that combining vitamin C with thiamine may have a neutral or even adverse effect in certain patient populations—for example, elderly individuals with multiple comorbidities [21].

Based on current evidence, thiamine appears to be safe and well tolerated at doses of approximately 200 mg intravenously every 12 h. No significant adverse effects have been observed, even with prolonged administration. Therefore, identifying the optimal target population—primarily patients with confirmed thiamine deficiency and determining the most appropriate timing for supplementation seems to be key. Current recommendations suggest targeted supplementation in patients with severe sepsis or septic shock, particularly in situations associated with an increased risk of vitamin deficiencies (e.g., gastrointestinal diseases, alcoholism, prolonged antibiotic therapy, or post-abdominal surgery). Given the limited availability of thiamine level testing in acute care settings, prophylactic administration in patients with a high likelihood of deficiency appears to be a rational approach.

Thiamine supplementation may represent a valuable component of adjunctive therapy in sepsis, particularly in patients with laboratory-confirmed deficiency of this vitamin and in cases of severe disease accompanied by lactic acidosis. However, further prospective, multicenter clinical trials with well-defined patient populations and appropriately selected endpoints are necessary to clearly establish the role of thiamine in standard sepsis management protocols.

### 3.3. Vitamin D

Vitamin D, although traditionally associated with the regulation of calcium-phosphate metabolism and bone mineralization, is increasingly described in the literature as a pleiotropic compound that plays a significant role in immune system function. Its active form, 1,25-dihydroxycholecalciferol (calcitriol), binds to vitamin D receptors (VDR), which are present on immune cells such as monocytes, macrophages, T and B lymphocytes, and dendritic cells. Through modulation of gene expression, vitamin D can influence the intensity of the inflammatory response, enhance the production of antimicrobial peptides such as LL-37, and reduce the secretion of pro-inflammatory cytokines. In the context of sepsis—a syndrome of systemic inflammatory response triggered by infection, leading to organ dysfunction and high mortality—vitamin D has become the subject of growing interest as a potential adjunctive therapy. In recent years, several important clinical studies have been published that shed new light on this relationship [22]. One of the most recent is a randomized clinical trial published by Ashoor and colleagues in 2024 in The Journal of Intensive Care Medicine [23]. This study evaluated the efficacy of two different doses of vitamin D_3_—low (5000 IU) and high (50,000 IU)—administered over seven days to patients with severe sepsis requiring mechanical ventilation in the intensive care unit. Patients receiving the higher dose showed significant improvements in clinical parameters. These included a reduction in procalcitonin levels, a decrease in SOFA scores—reflecting better organ function—an increase in LL-37 cathelicidin concentration, and a shorter duration of hospitalization. Importantly, the supplementation did not cause any toxic effects—over 90% of patients achieved a 25(OH)D level above 30 ng/mL, which is considered a therapeutic threshold [23]. Another retrospective study, published by Li et al. in 2025 in Frontiers in Cellular and Infection Microbiology [24], included over 19,000 patients with sepsis who were hospitalized in American intensive care units, with data sourced from the renowned MIMIC-IV database. The study demonstrated that patients who received vitamin D supplementation during hospitalization had significantly lower 28-day mortality rates, a reduced percentage of deaths in the ICU itself, and shorter hospital stays. The authors suggest that the observed effects may be due to vitamin D’s influence on the endothelial barrier, modulation of oxidative stress, and suppression of the transcription factor NF-κB, which is responsible for activating pro-inflammatory [24]. Despite these promising results, systematic reviews remain cautious in their conclusions. A study published in 2024 [25] analyzed thirteen randomized clinical trials aimed at evaluating the effectiveness of vitamin D in patients with sepsis or in critical condition. Only four of these studies demonstrated significant clinical benefits in terms of reduced mortality or improved patient outcomes. The review highlighted substantial variability in study protocols—differences included dosage (ranging from 4000 to as much as 600,000 IU), timing of therapy initiation, and baseline 25(OH)D levels. A key issue was that many studies failed to achieve vitamin D concentrations above 30 ng/mL, which may have considerably weakened the effectiveness of the intervention [25]. This issue was also thoroughly discussed in a review published in 2025, which highlighted that only approximately 41% of patients participating in clinical trials actually achieved therapeutic blood levels of vitamin D. The authors suggest that the lack of standardized dosing protocols and inadequate monitoring of 25(OH)D concentrations may have significantly contributed to the variability in outcomes [26]. Data from 2024–2025 provides increasingly strong evidence for the potential effectiveness of vitamin D in treating patients with sepsis, particularly those hospitalized in intensive care units. Clinical trials suggest that high doses of vitamin D may improve organ function, reduce inflammation, and shorten hospital stays, while supplementation may have a beneficial impact on mortality. At the same time, further well-designed randomized studies are necessary to establish standardized dosing protocols and to identify which patient groups benefit most from this intervention [27,28].

### 3.4. Folic Acid and Other B Vitamins

B vitamins, including thiamine (B1), riboflavin (B2), pyridoxine (B6), folic acid (B9), and cobalamin (B12), play essential roles in numerous metabolic processes that may be severely disrupted during sepsis. As cofactors for various mitochondrial enzymes, they are involved in energy metabolism, regulation of the Krebs cycle, amino acid and nucleic acid metabolism, and homocysteine turnover. Additionally, they exert antioxidant and immunomodulatory effects. In the face of the metabolic and inflammatory stress associated with sepsis, the demand for these vitamins increases significantly, and even subclinical deficiencies may contribute to the progression of multiorgan failure.

Thiamine holds a particularly important position among B vitamins, serving as an indispensable cofactor for pyruvate dehydrogenase and α-ketoglutarate dehydrogenase—enzymes critical for proper mitochondrial function and ATP production. In the context of sepsis, where aerobic metabolism is profoundly impaired and shifts toward glycolytic pathways (Warburg effect), thiamine deficiency may exacerbate lactic acidosis, mitochondrial dysfunction, and oxidative stress. Observational studies suggest that up to 30–35% of patients with severe sepsis exhibit markedly reduced plasma thiamine levels, and this deficiency is associated with poorer prognosis and higher mortality [29]. One of the most influential studies in this area is a post hoc analysis conducted by Vine et al. [17], which included data from two phase II randomized trials investigating thiamine monotherapy in patients with sepsis and septic shock. The study demonstrated that intravenous administration of thiamine (200 mg twice daily) significantly increased the likelihood of survival without the need for RRT, particularly in the subgroup of patients with biochemically confirmed thiamine deficiency. In this population, the proportion of patients who survived without requiring RRT was 61%, compared to 20% in the placebo group, suggesting a potential role for targeted therapy in high-risk populations [17]. It should be noted, however, that data from randomized trials remain inconclusive. A systematic review encompassing 16 RCTs and over 3000 patients did not demonstrate a statistically significant reduction in mortality or hospital length of stay in groups receiving thiamine. Benefits were observed only in subgroups treated with combination therapy (the so-called HAT protocol: hydrocortisone, ascorbic acid, and thiamine), where improvements in SOFA scores and reductions in lactate levels were reported—although these did not translate into significant clinical endpoints such as survival [30].

Folic acid (vitamin B_9_) plays a crucial role in DNA methylation, nucleotide synthesis, and homocysteine detoxification—processes essential for endothelial function, immune mechanisms, and resistance to oxidative stress. In the context of intensive care for sepsis, folate metabolism becomes profoundly disrupted, and plasma folate levels may serve both as prognostic indicators and modulators of disease progression.

An important finding comes from a prospective study published in 2022 by Gamarra-Morales et al., involving a group of 30 patients with septic shock [31]. The study found that although the average folate concentration was within the normal range, 21.4% of patients had elevated levels, while 14.2% presented with deficiencies. A significant correlation was observed between higher folate levels and 28-day mortality, as well as with prolonged mechanical ventilation, increased FiO_2_ requirements, and elevated fibrinogen concentrations [31]. The authors suggested that elevated folate levels in sepsis may not reflect a favorable nutritional status, but rather impaired cellular utilization or disrupted metabolic processing. Excess unmetabolized folic acid (UMFA) may exert harmful effects by intensifying oxidative stress and compromising endothelial barrier permeability—an especially critical concern in patients in severe condition. Data from the same analysis also revealed significant associations between folate levels and other clinical indicators, such as duration of mechanical ventilation, fibrinogen concentration, and oxygen requirements, further supporting their potential role as prognostic biomarkers in the course of septic shock [31]. From a clinical practice perspective, the issue of appropriate folate dosing in critically ill patients remains unresolved. Current reports indicate that both deficiency and excessive intake of folic acid may have negative clinical implications. In patients undergoing renal replacement therapy, significant folate losses are observed, which may lead to deficiency within the first days of hospitalization [32]. Studies suggest that daily intravenous supplementation with the active form of folate—calcium folinate—at a dose of 5 mg for 7 to 10 days can effectively normalize serum and erythrocyte levels of this vitamin [32]. In contrast, single high-dose administration protocols have proven significantly less effective in achieving sustained correction of deficiency.

In stable patients without absorption disorders, oral supplementation at a dose of 1 mg per day is recommended. However, in patients with severe sepsis, multiple organ failure, or those receiving parenteral nutrition, parenteral doses of around 5 mg/day appear to be more justified. These observations are consistent with current ESPEN and ASPEN guidelines, which emphasize the need to monitor B vitamin status in intensive care settings and to exercise caution in their supplementation—especially in cases of potential accumulation of inactive forms and metabolic insufficiency [33]. Folic acid remains a compound of significant, though still insufficiently understood, importance in the course of sepsis. Its role as a biomarker and potential therapeutic agent requires further investigation, including prospective studies involving larger patient groups that clearly define both supplementation criteria and clinical endpoints. Based on current knowledge, the use of moderate doses of folates in patients with documented deficiency appears justified, while caution is advised against uncontrolled supplementation—particularly in cases where concentrations of active metabolites are not being monitored.

Vitamin B12(cobalamin) plays a key role in the conversion of homocysteine to methionine, and its deficiency is associated with hyperhomocysteinemia, endothelial dysfunction, and increased lipid peroxidation. Observational studies suggest that in patients with severe sepsis, B_12_ levels are often reduced, which correlates with higher mortality, although no definitive intervention threshold has been established. Additionally, some studies indicate that elevated B_12_ levels may also be linked to poorer prognosis, particularly in populations with liver failure, which may result from limited cellular uptake and increased concentrations of the free fraction [34].

Recent systematic reviews published between 2023 and 2024 confirm that B vitamins may play an important role in the treatment of sepsis, but emphasize the need for personalized therapy. The authors point out that administration of these vitamins should be preceded by an assessment of baseline levels, and interventions should be tailored to the type of deficiency and the clinical context. Currently, there is a lack of clear guidelines regarding dosage, timing of therapy initiation, and the optimal administration regimen—both for thiamine and other B vitamins [35].

## 4. Nutritional Status and Microbiome Quality as Predictors of Mortality in Sepsis

The significant impact of nutritional components in sepsis is also demonstrated by studies evaluating the relationship between overall nutritional status and survival. The modified Glasgow Prognostic Score (mGPS) assesses the interplay between nutritional status and systemic inflammation based on serum albumin concentration and C-reactive protein (CRP) levels. The Prognostic Nutritional Index (PNI) combines serum albumin concentration and total lymphocyte count, providing a composite indicator of nutritional and immune status. The Controlling Nutritional Status (CONUT) score incorporates serum albumin, total lymphocyte count, and total cholesterol levels; this score has shown strong prognostic value for hospital mortality, risk of sepsis, and length of hospital stay among internal medicine patients [35]. The NUTRIC (modified Nutrition Risk in Critically Ill, mNUTRIC) score is the first validated nutritional index developed specifically for critically ill patients and serves as a reliable predictor of adverse clinical outcomes in various settings. The BAR (blood urea nitrogen-to-serum albumin ratio) combines markers of kidney function and protein status, representing a potentially more accurate predictor of outcomes in sepsis. These indices were evaluated by Toscano et al. in a cohort of 143 patients with sepsis or septic shock. A multivariable stepwise regression analysis was performed to identify factors independently associated with in-hospital mortality. Among the evaluated variables, the mNUTRIC score emerged as a significant independent predictor of death, with an area under the curve (AUC) of 0.814 and a cut-off value of 4.5 points as the optimal threshold for predicting mortality [36]. Although this study did not confirm the effect of vitamins discussed in the present article—being only one component potentially lacking in patients with poor nutritional status—it clearly indicates the significant role of nutritional factors in determining mortality in sepsis.

The second issue that needs to be addressed in this context is the impact of the microbiome. Gut dysbiosis is a key component of the pathogenesis and prognosis of sepsis. Patients with sepsis exhibit a marked decrease in microbiota diversity, loss of beneficial bacteria—particularly short-chain fatty acid (SCFA) producers such as butyrate-producing *Firmicutes*—and an overgrowth of pathobionts, mainly *Proteobacteria* and *Enterococcus* [37]. These alterations impair intestinal barrier integrity by reducing the expression of tight junction proteins (ZO-1, occludin) and SCFA production, promoting bacterial and endotoxin (LPS) translocation into the bloodstream [37]. As a result, inflammatory responses intensify, further aggravating the immune dysregulation characteristic of sepsis. A healthy gut microbiota, through metabolites such as butyrate, propionate, and acetate, plays a crucial role in modulating immune responses by promoting regulatory T-cell (Treg) differentiation, inhibiting NF-κB activation, enhancing interleukin-10 production, and supporting phagocytosis [37]. Consequently, loss of these functions leads to excessive inflammation and weakened antimicrobial defense. Dysbiosis may also affect hematopoiesis and neutrophil maturation, thereby disrupting systemic immune balance [37]. Broad-spectrum antibiotics, particularly those with anaerobic activity, exacerbate microbiota disturbances by eliminating beneficial taxa, decreasing SCFA levels, and allowing the overgrowth of resistant strains, which can increase susceptibility to opportunistic infections and worsen sepsis outcomes [37]. Clinical studies have explored biomarkers of gut barrier injury—such as intestinal fatty acid–binding protein, diamine oxidase, LPS, presepsin, and citrulline—as indicators of bacterial translocation and dysbiosis severity, though their diagnostic value remains to be validated. Characteristic “ICU enterotypes” have also been identified, correlating with disease severity and outcomes [37]. Therapeutic strategies aimed at restoring microbiota balance—including probiotics, prebiotics, synbiotics, postbiotics, fecal microbiota transplantation, and selective digestive decontamination—have shown mixed results, and some carry risks of pathogen transmission or bacteremia [37]. Although current evidence is mostly correlative and derived from preclinical or small-scale clinical studies, the microbiome’s role in sepsis appears to be fundamental. Future perspectives emphasize the personalization of sepsis therapy based on individual microbiome and metabolite profiles, coupled with rational antibiotic stewardship and nutritional strategies to support gut function and microbial resilience.

## 5. Study Limitations

Our review has several limitations that must be disclosed. Firstly, due to limited resources, we did not perform a systematic review. In cases where study results are inconsistent, conducting a meta-analysis would be advisable; however, this was not feasible because of substantial heterogeneity among studies, particularly in terms of the populations included. Secondly, a major challenge lies in the lack of translation of the demonstrated mechanisms into clear clinical outcomes. There are several possible explanations for this phenomenon. One possibility is that the underlying mechanism exerts a clinically meaningful effect only in cases of severe deficiency. Another explanation might be that supplementation was initiated too late to interrupt the ongoing inflammatory cascade. It is also plausible that the administered doses were insufficient to achieve a therapeutic effect. When interpreting such findings, it is crucial to consider the multifactorial nature of sepsis and the complexity of the host response. Differences in timing, dosage, baseline nutritional status, and patient characteristics can profoundly influence outcomes. Therefore, future research should not only aim to replicate mechanistic findings but also focus on well-designed, adequately powered clinical trials that account for these variables and assess both biochemical and clinical endpoints.

## 6. Conclusions

Sepsis, as a complex and dynamic disorder, remains one of the major challenges in modern intensive care, associated with high mortality, substantial treatment costs, and long-term clinical consequences. The severity of the initial organ failure, the underlying etiology, and the pathogen’s drug sensitivities remain the pivotal determinants of patient outcomes. Given the limited effectiveness of current therapeutic strategies, there is growing interest in adjunctive interventions aimed at supporting the disrupted metabolic and immunological homeostasis. Critically ill and septic patients frequently exhibit low plasma levels of essential vitamins (such as vitamin C, D, and B1). This depletion is a result of a combination of factors: increased metabolic demand, inflammatory redistribution, and often reduced intake or absorption. Crucially, observational data linking low vitamin levels to worse outcomes may reflect the severity of the illness itself (i.e., sicker patients have more deranged metabolism and depleted reserves) rather than demonstrating a true, modifiable cause of poor prognosis. Thus, while screening for deficiencies is important, a low plasma level may primarily serve as a prognostic marker rather than an indicator for blind supplementation.

Despite compelling mechanistic rationale (pleiotropic effects on mitochondrial function, antioxidant action, and immune modulation), robust, large-scale randomized controlled trial (RCT) evidence is still largely lacking or inconclusive for routine use:

- Vitamin C: Large-scale trials have not conclusively proven a mortality benefit, despite its well-documented antioxidant properties and microcirculatory effects.
- Vitamin D: Supplementation trials in sepsis and ICU settings have yielded mixed or inconclusive results. Benefits may be confined to specific, severely deficient subgroups, highlighting the need for a targeted approach.
- Vitamin B1: Benefits are primarily suggested in patients with documented deficiency, where intravenous administration may support metabolic balance, though overall RCT evidence remains inconclusive.
- Vitamins B9 and B12: Evidence is least robust; some studies suggest that measured plasma levels may be misleading due to rapid redistribution and altered binding proteins in the acute phase.

Current evidence is insufficient to recommend routine vitamin supplementation for all patients with sepsis. Furthermore, supplementing with high-dose vitamins blindly carries inherent risks (e.g., hypervitaminosis, drug interaction, metabolic derangements). Scientific evidence supports a shift from universal supplementation to a targeted and personalized approach. Future research should prioritize:

- Patient Selection: Focusing on high-risk subgroups, such as malnourished patients, the elderly, or those with pre-existing conditions (e.g., chronic liver disease or malabsorption) likely to entail deficiency.
- Tissue Sufficiency: Developing methods to measure tissue-level sufficiency rather than relying solely on potentially misleading plasma levels in the acute setting.
- Standardization: Implementing standardized dosing protocols and clearly defined clinical endpoints in well-designed multicenter trials to reliably assess the true role of specific vitamins as adjunctive therapy in sepsis.

## Data Availability

Not applicable.
